# Data-driven research with historically excluded groups: Towards a model for co-production and democratisation

**DOI:** 10.23889/ijpds.v8i2.2261

**Published:** 2023-09-14

**Authors:** Sarah McKenna, Elizabeth Nelson, Aideen Maguire

**Affiliations:** 1 Administrative Data Research Centre Northern Ireland, Belfast, United Kingdom; 2 Queen's University Belfast, Belfast, United Kingdom; 3 Ulster University, Belfast, United Kingdom

## Objectives

It is widely recognised that involving “experts by experience” from historically excluded groups (e.g. those with experience of care) is necessary to maximise research impact, ensure mutual benefit, and is central to the democratisation of data for research. We aim to develop a model of co-production in data-driven research.

## Methods

Public engagement in research generally, and data-driven research specifically, has increased significantly in the last decade. However, the co-production of data-driven research with the people behind the data is still rare. This pilot project was designed and delivered in partnership between Administrative Data Research Centre Northern Ireland (ADRC NI), Voice of Young People in Care (VOYPIC) and a group of care experienced young people. Through a range of methods (including meetings, a series of workshops and an internship for a care experienced young person at ADRC NI) we explored and tested opportunities for the co-production of research.

## Results

Led by the group of care experienced young people, we identified a range of entry points for the co-production of data-driven research. While some stages of the research process remained challenging for those with lived experience to penetrate, most notably the analysis of quantitative data in a secure environment, other stages of the research process were meaningfully co-produced. These include the prioritisation of research topics, the development of research questions, the interpretation of findings and the creation of lay language dissemination materials. Formal evaluation of the pilot is pending, but early benefits for all project partners include the development of new skills and knowledge, re-focused research priorities, and more accessible research outputs.

## Conclusion

Co-production of data-driven research with experts by experience is feasible and necessary for the democratisation of data. It delivers tangible benefits which amplify the positive impact of data-driven research. This session will share innovative practice, key learnings, and practical guidance for the co-production of data research with historically excluded groups.

